# The diagnostic and prognostic value of interleukin-6 in patients with soft tissue sarcomas

**DOI:** 10.1038/s41598-017-08781-6

**Published:** 2017-08-29

**Authors:** Tomohito Hagi, Tomoki Nakamura, Takahiro Iino, Takao Matsubara, Kunihiro Asanuma, Akihiko Matsumine, Akihiro Sudo

**Affiliations:** 1 Department of Orthopaedic Surgery, Mie University Graduate School of Medicine, Tsu, 514-8507 Japan; 2 Department of Orthopaedic Surgery, Fukui University Graduate School of Medicine, Fukui, 910-1104 Japan

## Abstract

The presence of systemic inflammation has been reported to be associated with poor prognosis in patients with soft tissue sarcoma (STS). The cytokine interleukin-6 (IL-6) has pleiotropic effects on various cell types in the tumor microenvironment. The aim of the present study was to determine whether serum IL-6 levels could be useful to assume the differentiation of benign soft tissue tumors from STS and to investigate the possible value of IL-6 for survival and oncological events in patients with STS. The medical records of 99 patients who underwent surgical resection were retrospectively reviewed. Serum IL-6 levels (median: 9.04 pg/ml) in patients with STS were statistically higher than those (3.31 pg/ml) in patients with benign soft tissue tumors. Our analyses confirmed that tumor size and IL-6 level were significant predictors of STS diagnosis. Next, we examined the relationship between IL-6 levels and survival in the 59 patients with STS. C-reactive protein levels, hemoglobin levels, and tumor grade were strongly correlated with IL-6 levels. Tumor grade and IL-6 level remained significant factors for survival and event-free survival. We suggest that measurement of IL-6 levels may be a useful method for identifying patients who are at a high risk of STS and tumor-related death.

## Introduction

Several prognostic factors have been defined in patients with soft tissue sarcoma (STS). Tumor size, depth, histological grade, and patient age are predictive factors for survival^1–3^. Recently, the presence of systemic inflammation has been reported to be associated with poor prognosis in patients with STS^4–7^. The cytokine interleukin-6 (IL-6) has pleiotropic effects on various cell types in the tumor microenvironment and is a regulator of the pro-oncogenic transcription factors NF-κB and STAT3^8^. Several studies have also demonstrated that high levels of IL-6 contribute to the clinical development of anemia and elevated C-reactive protein (CRP) levels^9–11^.

Previous reports indicate that cancer patients have increased IL-6 levels compared to healthy subjects and patients with benign tumors^7, 12, 13^. Rutkowski *et al*. reported that IL-6 levels in patients with STS were higher than those in healthy subjects^7^. IL-6 levels were also significantly associated with survival in studies of patients with several types of cancer^7, 12, 14, 15^.

Little is known about the association between IL-6 and STS. One study reported that STS patients with higher IL-6 levels had significantly poorer survival than those with lower IL-6 levels, although the analysis included patients with local recurrence and/or metastasis^7^. However, there have been no reports concerning the diagnostic value of IL-6 in the differentiation of benign soft tissue tumors and STS. The aim of the present study was to determine whether serum IL-6 levels could be useful to assume the differentiation of benign soft tissue tumors from STS. The secondary aim was to investigate the possible value of IL-6 for survival and oncological events in patients with STS.

## Results

### Patient, tumor, and treatment characteristics

The cohort included 57 men and 42 women with a mean age of 61.4 years (range, 10 to 88 years) at first presentation. Forty patients had benign tumors, which were histologically classified as follows: lipoma (n = 18), schwannoma (n = 10), desmoid/fibroma (n = 5), and other tumors (n = 7). The remaining 59 patients had STS, histologically classified as follows: well-differentiated liposarcoma (n = 14), undifferentiated pleomorphic sarcoma (n = 10), myxofibrosarcoma (n = 9), de-differentiated liposarcoma (n = 7), leiomyosarcoma (n = 5), malignant peripheral nerve sheath tumor (n = 4), myxoid liposarcoma (n = 2), synovial sarcoma (n = 2), and other high-grade sarcomas (n = 6). According to the FNCLCC histological grading system, 16 sarcomas were grade 1, 20 sarcomas were grade 2, and 23 sarcomas were grade 3. All patients underwent surgical tumor resection. Adjuvant radiotherapy and chemotherapy were performed in 6 patients and 11 patients (neo-adjuvant and adjuvant 7, adjuvant alone 4), respectively. Adjuvant radiotherapy was considered for patients with inadequate surgical margins. Chemotherapy was considered for tumors close to vessels, nerves, or bones. We also performed chemotherapy for young patients. In the 59 patients with STS, the median CRP level was 0.19 mg/dl (mean: 1.36 mg/dl, range: 0.01–15.87 mg/dl), and the median hemoglobin (Hb) level was 13.9 g/dl (mean: 13.6 g/dl, range: 8.3–17.9 g/dl).

### Serum IL-6 levels and related clinicopathological variables in patients with soft tissue sarcomas and benign soft tissue tumors

The clinicopathological features of the 99 patients with soft tissue tumors are detailed in Table 1. Patients with STS were significantly older than those with benign soft tissue tumors (*p* = 0.0003). Age exhibited a weak-to-low correlation with IL-6 level (Spearman ρ = 0.35, *p* = 0.0004).

**Table 1:** The association between IL-6 and patients’ characteristics in soft tissue tumors.

Variables		Benign tumors	STS	p value
		(n = 40)	(n = 59)	
Age	Mean (years)	60	66	0.0003*
Gender	Male	25	32	0.54
	Female	15	27	
Tumor depth	Superficial	7	10	0.99
	Deep	33	49	
Tumor size	Mean (cm)	6	10	0.0006*
	>5 cm	25	50	0.02
	<5 cm	15	9	
IL-6	Mean (pg/ml)	3.89	35.9	<0.0001*

**Figure 1:**
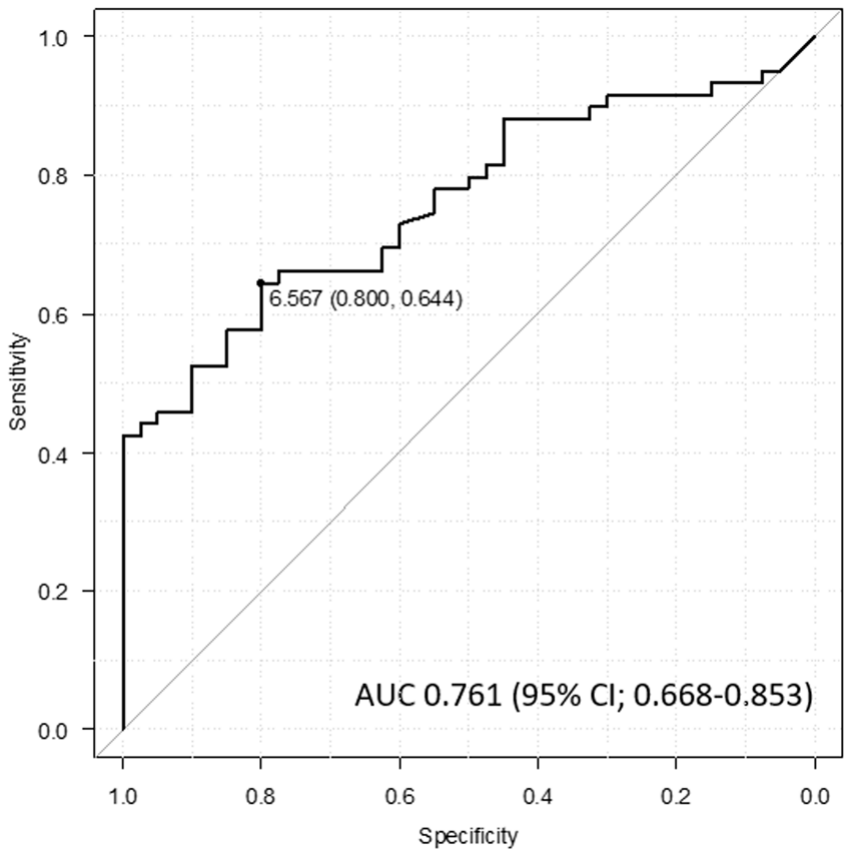
ROC curve shows the appropriate threshold of the IL-6 level for identifying soft tissue tumor patients at risk of developing STS.

The median IL-6 level was 9.04 pg/ml (mean: 35.9 pg/ml, range: 0–552.6 pg/ml) in patients with STS and 3.31 pg/ml (range = 0–13.5 pg/ml, mean = 3.89 pg/ml) in patients with benign soft tissue tumors. The IL-6 levels in patients with STS were statistically higher than those in patients with benign soft tissue tumors (odds ratio [OR]: 1.17, *p* < 0.0001). A logistic regression model confirmed that tumor size (OR: 1.17, *p* = 0.003) and IL-6 level (OR: 1.05, *p* = 0004) remained significant predictors of STS diagnosis (Table 2). In the ROC analysis, a serum IL-6 level of 6.567 pg/ml was found to be the optimal threshold for identifying patients at risk for a diagnosis of STS. The area under the curve (AUC) was 0.761 (95% confidence interval [95% CI]: 0.668–0.853) (Fig. 1). An elevated IL-6 level (>6.567 pg/ml) was observed in 37 of 59 patients with STS and 8 of 40 patients with benign soft tissue tumors. At this threshold, serum IL-6 level exhibited a sensitivity and specificity of 64.4% and 80.0%, respectively, for assuming STS.

**Table 2:** Logistic regression analysis for prognostic factors for STS.

Variables		Odd ratio (95% CI)	p value
Age	years	1.05 (1–1.10)	0.03
Tumor size	cm	1.17 (1.06–1.30)	0.003
IL-6	pg/ml	1.17 (1.05–1.30)	0.004

**Figure 2:**
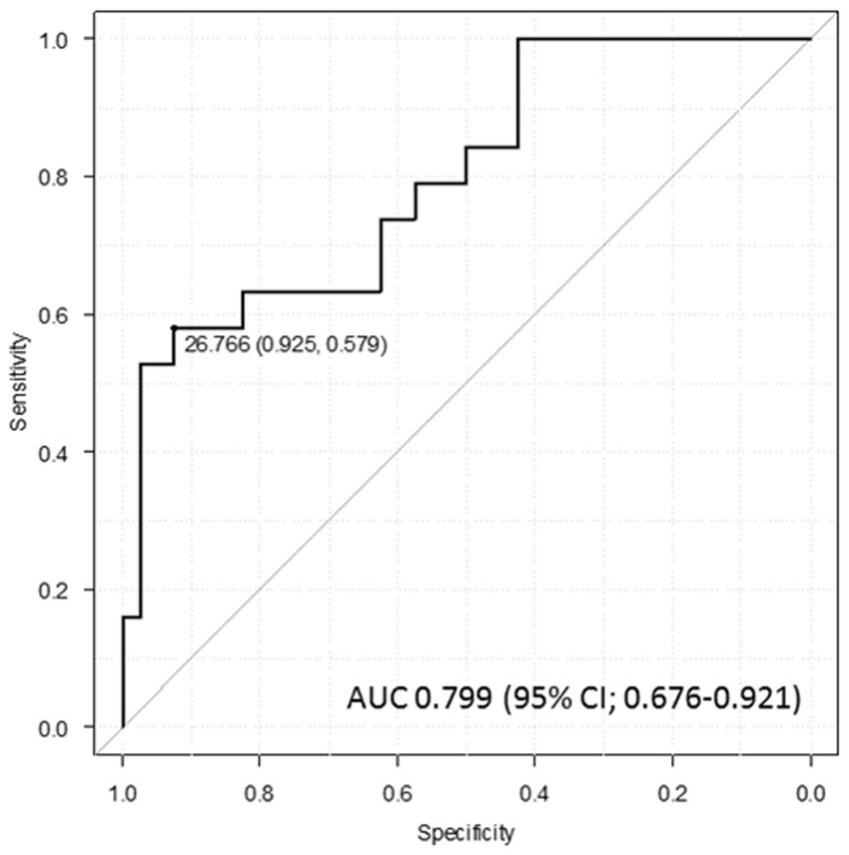
ROC curve shows the appropriate threshold of anemia to identify if patients are at risk from oncological event at 2 years.

Next, we examined the relationship between IL-6 levels and survival in the 59 patients with STS. This group included 32 males and 27 females with a mean age of 66 years (range, 24 to 88 years). The mean follow-up was 33.9 months (range, 2.4 to 75.9 months). The associations between IL-6 levels and clinicopathological variables in patients with STS are shown in Tables 3 and 4. CRP levels were strongly correlated with serum IL-6 levels based on the Spearman rank correlation test (Spearman ρ = 0.72, *p* < 0.0001). Furthermore, Hb levels (Spearman ρ = −0.40, *p* = 0.0015) and tumor histological grade (*p* < 0.0001) were associated with IL-6 levels. In the ROC analysis, a serum IL-6 level of 26.7 pg/ml was found to be the optimal threshold for identifying patients at risk for oncological events at 2 years. The AUC was 0.799 (95% CI: 0.676–0.921) (Fig. 2). We used the value 26.7 pg/ml to evaluate the prognostic value of IL-6 for oncological events in patients with STS.

**Table 3:** Correlation of IL-6 with clinical variables using Spearman correlation test.

Variables		Spearman ρ	p value
Age	years	0.1	0.45
Tumor size	cm	0.0016	0.99
CRP	mg/dl	0.72	<0.0001
Hb	g/dl	−0.4	0.002

**Table 4:** The association between IL-6 and patients’ characteristics in STS.

Variables		N	Mean levels of IL-6 (pg/ml)	p value
Gender	Male	32	9.98	0.65
	Female	27	8.51	
Tumor depth	Superficial	10	8.06	0.58
	Deep	49	10.44	
Tumor grade	G1	16	4.01	<0.0001*
	G2	20	6.71	
	G3	23	23.76

### Overall survival (OS), event-free survival (EFS) and their prognostic factors in patients with STS

As of December 2016, 30 of the 59 patients demonstrated continuous disease-free survival, 10 had no evidence of disease, 3 were alive with disease, 15 had died of disease, and one had died of other causes. The 5-year overall survival rate was 59.9% (95% CI: 32.6–79%). Local recurrence developed in 11 patients and metastasis developed in 20 patients. The 5-year event-free survival rate was 44% (95% CI: 25.7–60.9%).

**Figure 3:**
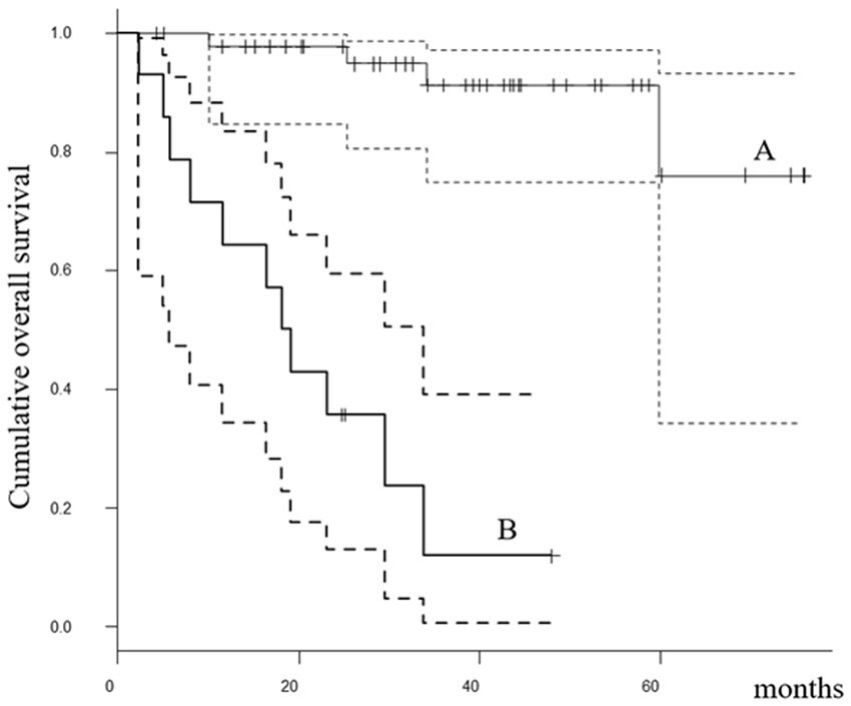
The Kaplan-Meier curves show the overall survival of 59 soft tissue sarcoma patients: (**A**) patients with IL-6 < 26.7 pg/ml; (**B**) patients with IL-6 ≥ 26.7 pg/ml.

Patients with elevated serum IL-6 levels (≥26.7 pg/ml) before initial treatment had poorer OS than patients with lower IL-6 levels (<26.7 pg/ml). The estimated OS at 2 and 5 years was 35.7% (95% CI: 13.0–59.4%) and not applicable, respectively, for patients with higher IL-6 versus 97.7% (95%CI: 84.6–99.7) and 76.1% (95% CI: 34.3–93.2), respectively, for those with lower IL-6 (*p* < 0.0001, Fig. 3). The univariate analysis also revealed that tumor histological grade was a predictive factor for OS (*p* = 0.006) (Table 5). In the univariate Cox proportional hazards analysis, pretreatment IL-6 level was an independent prognostic factors of OS in the 59 patients with STS (p = 0.0007) (Table 6). In multivariate analysis, tumor grade and IL-6 level remained significant prognostic factors of survival (p = 0.02 and p = 0.02, respectively) (Table 7).

**Table 5:** Univariate overall survival and event-free survival analysis in 59 patients with STS.

Variables	n	Overall survival		p value	Event-free survival		p value
		2-year	5-year		2-year	5-year	
Age							
>66 years	32	75.9%	67.4%	0.5	63.1%	34.5%	0.53
<66 years	27	88.3%	57.7%		68.9%	54.8%	
Gender							
Male	32	85.5%	75%	0.33	65.1%	44.3%	0.95
Female	27	77.6%	34.1%		66.5%	NA	
Tumor depth							
Superficial	10	80%	80%	0.54	70%	70%	0.36
Deep	49	82%	51.9%		64.7%	36%	
Tumor size							
>10 cm	26	82.8%	NA	0.82	69.4%	NA	0.31
<10 cm	33	81%	61.5%		62.5%	39.7%	
Tumor grade							
G1	16	100%	100%	0.0006	100%	80%	0.008
G2	20	90%	77.50%		62.3%	24.9%	
G3	23	64.30%	34.60%		47.1%	33.5%	
IL-6							
>26.7 pg/ml	14	35.70%	NA	<0.0001	19%	NA	<0.0001
<26.7 pg/ml	45	97.70%	76.10%		80.3%	52.7%

**Table 6:** Univariate cox proportional hazard analysis for overall survival and event-free survival in 59 patients with STS.

Variables		Overall survival	p value	Event-free survival	p value
		HR (95%CI)		HR (95%CI)	
Age	years	1.049 (0.998–1.102)	0.06	1.03 (0.992–1.068)	0.12
Tumor size	cm	1.014 (0.943–1.089)	0.71	0.972 (0.916–1.033)	0.37
IL-6	pg/ml	1.005 (1.002–1.008)	0.0007	1.006 (1.003–1.009)	0.0003

**Table 7:** Multivariate analysis of prognostic factors of STS for overall survival and event-free survival.

Variables		Overall survival	p value	Event-free survival	p value
		HR (95%CI)		HR (95%CI)	
Tumor grade	Grade	3.505 (1.273–9.648)	0.02	1.005 (1.001–1.008)	0.005
IL-6	pg/ml	1.003 (1.273–9.648)	0.02	2.152 (1.181–3.92)	0.01

**Figure 4:**
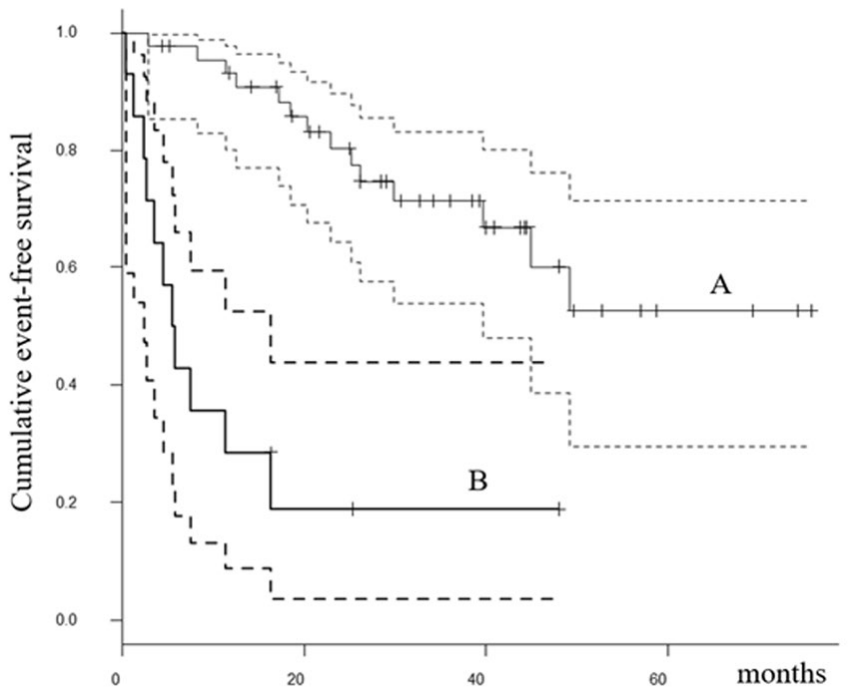
The Kaplan-Meier curves show the event-free survival of 59 soft tissue sarcoma patients: (**A**) patients with IL-6 < 26.7 pg/ml; (**B**) patients with IL-6 ≥ 26.7 pg/ml.

Patients with elevated serum IL-6 levels (≥26.7 pg/ml) before initial treatment had poorer event-free survival than patients with lower IL-6 levels (<26.7 pg/ml). The estimated EFS at 2 and 5 years was 19.0% (95% CI: 3.6–43.7%) and not applicable, respectively, for patients with higher IL-6, versus 80.3% (95% CI: 64.4–89.7%) and 52.7% (95% CI: 29.6–71.3%), respectively, for patients with lower IL-6 (*p* < 0.0001, Fig. 4). The univariate analysis also revealed that tumor histological grade was a prognostic factor of events (*p* = 0.0084) (Tables 5 and 6). On multivariate analysis, tumor grade and IL-6 level remained significant factors of events (p = 0.01 and p = 0.005, respectively) (Table 7).

## Discussion

The present study revealed that IL-6 levels may be used as an additional marker for the differential diagnosis of STS. Furthermore, IL-6 levels were associated with OS and EFS in STS patients with no metastatic disease at initial presentation.

IL-6 is produced in a variety of cells, including fibroblasts, endothelial cells, and macrophages^8, 12, 16^. IL-6 can also be produced by cancerous cells and tissues^17, 18^. Although we did not evaluate the tissue expression of IL-6, the serum expression of IL-6 does not necessarily correlate with its tissue expression^19, 20^.

Although soft tissue masses can be readily identified based on imaging characteristics, many STS remain indeterminate and require a biopsy to determine histological diagnosis and tumor grade. The identification of additional differential diagnostic markers that are accurate and readily available has the potential to aid in the clinical management of patients with STS. We previously reported that high-sensitivity CRP was a useful measure for the differentiation of benign and malignant STS^21^. In the present study, we found that elevated serum IL-6 level was also associated with the presence of STS and therefore could be used as an additional marker for the differential diagnosis of STS. However, sensitivity and specificity were not sufficient for accurate differentiation. We recommend assessing several sources of information, such as radiography, blood markers, and clinical behavior, when considering biopsy. An IL-6 level of 6.567 pg/ml was the optimal threshold for identifying patients with STS in the present study. Rutkowski *et al*. reported that IL-6 levels in healthy subjects ranged from 0.5 to 6 pg/ml, which was similar to those with benign tumors in present study (median = 3.31 pg/ml, mean = 3.89 pg/ml).

We also examined the utility of IL-6 as a prognostic factor to predict survival and events in 59 STS patients. IL-6 levels were related to tumor histological grade. We found that elevation of serum IL-6 was associated with decreased OS and EFS in patients with STS, suggesting that IL-6 elevation before treatment may be related to aggressive tumor behavior. In the present study, the IL-6 level of 26.7 pg/ml was found to be the optimal threshold with a reliable AUC using ROC analysis. Rutkowski *et al*. reported that STS patients with higher IL-6 levels had poorer survival compared to those with lower IL-6 levels^7^. Based on data from healthy subjects, these authors established an IL-6 cut-off value of 2.4 pg/ml, which is lower than the threshold determined in our study^7^. We calculated the threshold of 26.7 pg/ml using data from patients with STS. Therefore, we believe this value may reflect the risk of oncological events and death in patients with STS more accurately. However, a large-scale study is necessary to validate this observation. Several relevant prognostic factors have been defined for STS. Previous studies indicate that tumor size, depth, and histological grade are prognostic factors of survival and oncological events^1–3, 22^. We found that tumor grade was also a prognostic factor for survival and oncological events in addition to IL-6.

In the present study, IL-6 levels were correlated with CRP and Hb levels. In liver cells, IL-6 is a strong inducer of hepcidin, an iron-regulatory hormone that is responsible for inflammation-induced iron disutilization, resulting in the anemia associated with malignant tumors^9^. Furthermore, IL-6 is known to induce the production of CRP in hepatocytes^11^. Our results suggest that CRP levels may be a surrogate marker for IL-6 because these markers were strongly correlated. CRP is familiar to physicians and both easy and inexpensive to measure compared to IL-6. CRP and Hb have been associated with prognosis for local control, events, and survival in patients with STS^4–6, 23, 24^.

There are a few limitations to the present study. First, the presence of systemic disease may be associated with higher levels of inflammatory markers. Although we performed blood examinations and computed tomography scans of the lungs, abdomen, and pelvis according to the American Joint Committee on Cancer staging system, some inflammatory conditions may not have been detected. We could not analyze the relationship between IL-6 and each histological grade and classification because of small number of patients. The retrospective design of the study is another limitation. A prospective study is necessary to validate our results because our cohort included patients who had and had not been administered adjuvant therapy. However, we believe that the measurement of IL-6 levels may be a useful method of recognizing patients who are at greater risk of STS and tumor-related death.

In conclusion, we found that higher serum IL-6 level was associated with the presence of STS as opposed to benign tumors. These results suggest that inflammatory blood markers may be useful a diagnostic tool for the differentiation of benign and malignant soft tissue tumors. Furthermore, multivariate analysis revealed that IL-6 elevation before initial treatment was a significant prognostic factor of poorer OS and EFS among STS patients.

## Patients and Methods

### Patients

The medical records of 99 patients who underwent surgical resection between January 2008 and December 2015 were retrospectively reviewed, including 40 patients with benign soft tissue tumors and 59 patients with STS. Patients who presented with recurrent disease or/and metastasis or who were referred for additional resection after a previous inadequate excision were excluded from the study. Patients with an obvious history of myocardial infarction or infectious disease were also excluded. Histopathological diagnosis and tumor grade, determined using the French Federation of Cancer Centers Sarcoma Group (FNCLCC) system, were reviewed in all patients and confirmed by independent pathologists. Blood samples from all patients were obtained within one month prior to initial treatment. All samples were stored at −80 °C until measurement. Samples underwent centrifugation at 1,000 × g for 15 min and were measured using the R&D Systems™ Quantikine® ELISA kit (Minneapolis, USA). The present study was approved by the Ethics Committee of Mie University Hospital. Written informed consent was obtained from all patients. The design and procedures of the study were carried out in accordance with the principles of the Declaration of Helsinki.

### Statistical Analysis

Statistical associations between clinicopathological factors were evaluated using the Mann-Whitney *U*-test and Kruskal Wallis test for quantitative data and the chi-squared test for qualitative data. Correlations between IL-6 levels and clinical characteristics were tested using Spearman’s rank-order correlation analysis. A significant Spearman ρ implied a correlation in the population. Receiver operating characteristic (ROC) analysis was performed to determine the threshold IL-6 level for the assessment of STS risk. Survival time was defined as the interval between the date of initial treatment for the primary tumor and the last date when the patient was documented to be alive or the date of death. Survival curves were constructed using the Kaplan-Meier method. The log-rank test was used to compare the survival of patients with high versus low IL-6 levels. A multivariate analysis was performed using a Cox proportional hazards model using the significant predictors identified in the univariate analysis as variables. A value of *p* < 0.05 was considered to be significant in all statistical analyses. All statistical analyses were performed with the EZR graphical user interface (Saitama Medical Center, Jichi Medical University, Saitama, Japan) for R (The R Foundation for Statistical Computing, Vienna, Austria), which is a modified version of R Commander designed to add statistical functions frequently used in biostatistics.
